# Multiple Recurrent Stent Thrombosis in a Patient with Coexisting Clopidogrel Resistance and Increased Anticardiolipin Antibodies: A Case Report

**DOI:** 10.1155/2010/974149

**Published:** 2010-06-13

**Authors:** Erik H. Middlebrooks, Mukta Panda

**Affiliations:** College of Medicine, University of Tennessee, Chattanooga, TN 37403, USA

## Abstract

The antiphospholipid syndrome (APS) is a common cause of both arterial and venous thrombosis. While studies exist demonstrating the role of APS in coronary artery bypass graft failure, its role in stent thrombosis is less clearly documented. Also, a literature search of PubMed did not reveal any articles regarding the coexistence of clopidogrel resistance and APS despite increasing awareness of resistance to clopidogrel treatment. We present a case of a 59-year-old male having recurrent myocardial infarction after subacute restenosis of multiple drug-eluting stents despite anticoagulant therapy. The patient had in-stent thrombosis of seven drug-eluting stents in a course of eight days. He was subsequently found to have mild elevation of IgG anticardiolipin (aCL) antibody titers and resistance to clopidogrel. Long-term anticoagulation with a combination of low-molecular-weight heparin, clopidogrel, and aspirin has been effective. While the patient's aCL titer level was not elevated above the level required by the current diagnostic criteria for APS, we believe that this patient suffers from the antiphospholipid syndrome. We will discuss some of the controversies surrounding the diagnosis of APS as well as appropriate treatment and recognition of the coexistence of APS and clopidogrel resistance in patients with stent thrombosis.

## 1. Introduction

The antiphospholipid syndrome (APS) is an increasingly recognized cause of thrombotic events in patients. Antiphospholipid syndrome is the most common acquired cause of venous and/or arterial thrombosis and is most often associated with coronary, cerebrovascular, retinal artery, placental, and deep vein thrombosis [1]. There is evidence that anticardiolipin antibody titers have a direct correlation with the incidence of coronary artery bypass graft occlusion [2]; however, there are few reports of acute stent thrombosis in the literature [3–5]. 

Acute drug-eluting stent thrombosis has also been associated with clopidogrel resistance [6]. Studies have shown significant increases in major adverse cardiac events after coronary intervention in patients treated with clopidogrel who have decreased platelet aggregation [7]. The implications of this phenomenon on patients with coexisting hypercoagulable disorders are potentially life-threatening and should be recognized early.

We present a case of multiple acute stent thromboses associated with clopidogrel resistance and elevations in anticardiolipin antibody levels, which are insufficient to meet the diagnostic criteria of APS. These patients may be part of the clinical continuum of “seronegative” APS patients [8] and those who meet the laboratory criteria. We hope to illustrate potential weaknesses of the current diagnostic criteria and the importance of recognizing clopidogrel resistance in hypercoagulable patients to increase detection of at-risk patients.

## 2. Case Presentation

A 59-year-old male presented to the emergency room with sudden, sharp, left-sided chest pain without radiation. He has a long-standing history of hypertension, hyperlipidemia, and coronary artery disease. He had a previous myocardial infarction approximately 10 years prior for which he had a bare-metal stent placed in his right coronary artery. The patient has a 50-pack-year history of smoking, but he quit 10 years ago after his first myocardial infarction. He does not drink alcohol or use illicit drugs. His family history is only positive for hypertension. He was compliant with his medications which were lisinopril, amlodipine, simvastatin, metoprolol, omeprazole, and a 325 mg aspirin. 

He was found to have a normal complete blood count, with a hemoglobin of 13.8 g/dL, hematocrit of 40%, white blood cell count of 6,700/mm^3^, and platelet count of 266,000/mm^3^. His coagulation studies revealed a partial thromboplastin time (PTT) of 27 sec (normal 22.1–37.6 sec) and an initial prothrombin time (PT) of 15.7 sec (normal 11.9–14.9 sec) with an INR of 1.22. The results of a complete electrolyte panel, liver function studies, and lipid panel were all within normal limits except for an HDL of 26 mg/dL (normal 40–60 mg/dL). Cardiac enzymes were initially normal but continued to increase to a CK-MB Index of 8.6 (>4 consistent with myocardial infarction), CPK of 971 U/L (30–200 U/L), and Troponin-I of 17.49 ng/mL (normal <1.5 ng/mL).

The patient underwent cardiac catheterization and had 60–75% restenosis of the mid right coronary artery (RCA) and 75% stenosis of the left circumflex artery. There was placement of a 2.75 × 32 mm and a 2.75 × 12 mm Taxus drug-eluting stent (Boston Scientific Corporation) in the RCA and a 2.75 × 24 mm Taxus drug-eluting stent in the left circumflex. Excellent flow was noted and 0% residual stenosis remained after the stenting. The patient received 600 mg clopidogrel after the procedure. 

Approximately 90 minutes later, the patient developed severe chest pain and ST-segment elevation in the inferior leads on a 12-lead EKG. He was immediately moved to the catheterization suite for repeat cardiac catheterization. There were occlusions of both the RCA and left circumflex artery stents. After thrombectomy, the patient received heparin and eptifibatide. Another 2.5 × 24 mm Taxus drug-eluting stent was placed in the left circumflex in addition to previous stent. The ST-segment returned to baseline and the patient was without chest pain. The patient was continued on clopidogrel, aspirin, and eptifibatide for the following four days and then discharged home.

**Figure 1:**
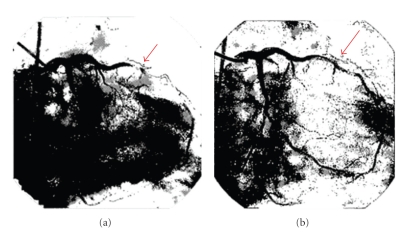
Left circumflex artery before (a) and after (b) stent placement during the patient's third cardiac catheterization.

**Figure 2:**
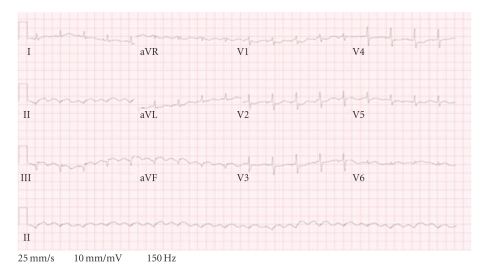
EKG, showing ST-segment elevation in contiguous inferior leads (II, III, aVF), taken after discontinuation of heparin drip and before undergoing coronary artery bypass grafting.

**Figure 3:**
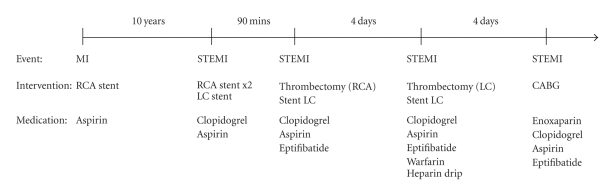
Timeline of events, interventions, and medications.

The patient returned to the emergency room the same day as discharge complaining of severe chest pain. He again had ST-segment elevation and underwent cardiac catheterization. There was 100% restenosis of the left circumflex artery (Figure 1), but good inflow of the RCA. Thrombectomy was performed, and a 2.5 × 24 mm Taxus stent was deployed in the left circumflex. Hypercoagulable laboratory studies were drawn and thromboelastograph demonstrated clopidogrel resistance in the patient, with platelet inhibition of 4%. Therapy was started with eptifibatide, clopidogrel, aspirin, warfarin, and a heparin drip. After four days, the INR was 2.41 and the heparin drip was discontinued. Soon afterwards, the patient developed recurrent chest pain (Figure 2) and was taken back to the cardiac catheterization lab. Both the left circumflex artery and RCA stents were re-stenosed. He was referred to surgery for emergent coronary bypass graft. The patient was stabilized and discharged home on low-molecular-weight-heparin, aspirin, and clopidogrel. A complete timeline of events is shown in Figure 3.

After an extensive hypercoagulable work-up, the patient tested negative for antinuclear antibody (ANA), IgA/IgM anticardiolipin antibody, homocysteine, IgG/IgA/IgM *β*2 glycoprotein, Protein C deficiency, Protein S deficiency, anti-thrombin III deficiency, Factor V Leiden, and Prothrombin 20210A. A dilute Russell Viper venom time was 35 sec (normal <43 sec). The only abnormal laboratory values were an IgG anticardiolipin antibody of 12 (normal <10) and a dilute prothrombin time (dPT) of 63.60 (normal 0–56.6 sec), with no lupus anticoagulant detected.

In follow-up with the patient, he has remained on low-molecular-weight heparin, aspirin, and clopidogrel for 6 weeks. He has had no chest pain or other known thrombosis. His repeated laboratory studies show an undetectable level of IgG anticardiolipin antibody as well as undetectable levels of IgA/IgM anticardiolipin antibody or lupus anticoagulant.

## 3. Discussion

Clopidogrel resistance is an emerging topic in the literature. It is unclear whether the phenomenon is merely a preexisting variability in platelet response to ADP or a true decrease in response to clopidogrel [9]. Regardless of the pathophysiology, studies have shown a significant increase in major adverse cardiac events after coronary intervention in patients treated with clopidogrel who have decreased platelet aggregation [7]. Nonresponsiveness to clopidogrel also has a strong correlation with drug-eluting stent thrombosis [6]. However, varying definitions of “nonresponsiveness” have made it difficult to know the true incidence of the condition and the direct correlation with clinical outcomes.

There is evidence that increased dosing of clopidogrel reduces the incidence of platelet nonresponsiveness [10] and significantly increases clinical outcomes [11, 12]. The Clopidogrel Optimal Loading Dose Usage to Reduce Recurrent Events/Optimal Antiplatelet Strategy for Interventions (CURRENT/OASIS-7) trial did demonstrate a 30% reduction in stent thrombosis and a 22% reduction in myocardial infarction in patients treated with high-dose clopidogrel as compared to the current dosing guidelines. The high-dose arm of the study consisted of a 600 mg loading dose of clopidogrel followed by 150 mg daily for seven days [13]. In addition, in vitro studies have also shown that treatment with eptifibatide and 600 mg clopidogrel added a greater than or equal to 2-fold increase in platelet inhibition compared to 600 mg or 300 mg clopidogrel alone [14]. The implications this will have on treatment for patients with hypercoagulable conditions remain to be seen.

IgG anticardiolipin antibodies (IgG aCL) have been linked to coronary artery thrombosis. Up to 38.3% of patients with positive titers for IgG aCL have coronary, carotid, or peripheral arterial thrombosis. The incidence is more than doubled compared to patients with the classic lupus anticoagulant [15]. Coronary artery bypass graft (CABG) occlusion is also directly correlated with aCL titers [2]; however, PubMed search of the literature reveals few previous case reports of APL-associated recurrent stent thrombosis [3–5]. Unfortunately, it is unknown whether these patients also demonstrated resistance to clopidogrel. These case reports, combined with the aforementioned studies, suggest a correlation in antiphospholipid syndrome and recurrent stent thrombosis.

Similar to previous case reports, our patient has a strong propensity to reocclude coronary stenting. The current consensus criteria for the diagnosis of APS due to IgG anticardiolipin include clinical evidence of thrombosis in conjunction with an aCL titer persistently elevated >99th percentile, or >40 GPL or MPL [16]. Given the patient's clinical manifestations, increased INR, and elevated IgG aCL, which is not sufficiently elevated to meet the diagnostic criteria, we believe that the patient should be classified as having antiphospholipid syndrome associated with aCL. The absence of any other abnormal hematological labs is consistent with this diagnosis. The patient will receive long-term fixed-dose anticoagulation; therefore, the absence of abnormal labs during follow-up may be expected because therapy has been shown to decrease anticardiolipin antibody levels, in particular aspirin [17].

There is controversy surrounding the diagnostic criteria for APS [18]. There have been reports of patients presenting with thrombotic episodes and normal antibody titers who were found to be positive 2–7 months later [8]. These “seronegative” patients suggest that current diagnostic criteria are insufficient to diagnose all affected patients. There also exists controversy surrounding the laboratory tests themselves. A study from the First French Anticardiolipin Standardization Workshop demonstrated a variation of greater than 70% in IgG aCL between laboratories [19].

We believe that this case represents a patient with APS who is excluded by the current consensus diagnostic criteria. An expansion of the criteria should be considered to include patients with lower antibody titers (cutoff less than the current 40 GPL or MPL) in light of evidence suggesting a prothrombotic predisposition in these patients. Also, search of PubMed reveals no literature regarding the coexistence of prothrombotic conditions and clopidogrel resistance. However, we believe tat recognition of this coexistence could have dramatically altered our patient's clinical course. 

Despite the previously mentioned studies regarding improved outcomes in clopidogrel-resistant patients with altered drug regimens, we believe this to still be insufficient treatment for patients with a coexistent hypercoagulable disorder. Awareness of the possibility of prothrombotic conditions coexisting with drug resistance in patients undergoing clopidogrel therapy could have a dramatic reduction in their morbidity and mortality. Although formal studies are lacking at this time, we suggest the use of antiplatelet agents in combination with unfractionated or low-molecular-weight heparin to prevent recurrent stent thrombosis in patients with antiphospholipid syndrome or increased anticardiolipin antibody levels based on our experiences.
